# Nutrient-rich feed supplementation accelerates recovery of body condition and increases calf birth weight in Bali cattle affected by foot and mouth disease

**DOI:** 10.5455/javar.2025.l877

**Published:** 2025-03-24

**Authors:** Yusuf Akhyar Sutaryono, Dahlanuddin Dahlanuddin, Ryan Aryadin Putra, Adji Santoso Dradjat, Syamsul Hidayat Dilaga, Suhubdy Suhubdy, Sukarne Sukarne, Dedi Supriadi

**Affiliations:** 1 Laboratory of Forage Production, Faculty of Animal Science, University of Mataram, Mataram, Indonesia; 2 Laboratory of Ruminant/Herbivore Nutrition, Faculty of Animal Science, University of Mataram, Mataram, Indonesia; 3 Laboratory of Animal Reproduction and Breeding, Faculty of Animal Science, University of Mataram, Mataram, Indonesia

**Keywords:** Bali cattle, birth weight, body condition, foot and mouth disease, supplementation

## Abstract

**Objective:**

This study aimed to evaluate the effects of feed supplementation on the body condition of cows suffering from foot and mouth disease (FMD) and the birth weight of their calves.

**Materials and Methods:**

The study involved 30 pregnant cows (6–7 months gestation), divided into five groups of six cows each. Groups A, B, C, D, and group E were supplemented with concentrates at levels of 0%, 0.2%, 0.4%, 0.6%, and 0.8% of body weight, respectively. The concentrates comprised cassava peels (60%), corn mill (40%), urea (3% of total feed needed), and minerals (25% of total feed needed).

**Variables:**

Following the onset of FMD symptoms, observations were made on the duration of hypersalivation, nasal discharge, snout and nose erosion, tongue and lip erosion, foot swelling, refusal to eat, and return to normal feed consumption.

**Results:**

The results showed that feed supplementation accelerated recovery such as time from hypersalivation, nasal discharge, muzzle erosion, and tongue (*p <* 0.05). However, no significant difference in interdigital wounds and duration of reluctance to eat due to FMD. Providing nutrient-rich feed also increases the body condition scores after FMD infection and the calf birth weight (*p <* 0.05) of Bali cattle.

**Conclusion:**

It was concluded that nutrient-rich feed supplementation accelerates cow recovery from FMD, maintains body condition, and increases the calf birth weight of Bali cattle.

## Introduction

Foot and mouth disease (FMD) is a highly contagious disease that causes significant economic losses globally [1–3]. It is caused by an RNA virus of the *Picornaviridae *family of the genus *Aphtovirus *[4]*.* FMD first entered Indonesia from the Netherlands in 1887, with the last outbreak reported in 1983. Following mass vaccination efforts in 1986, Indonesia was declared free of FMD by the government in 1990. However, in May 2022, a highly contagious outbreak occurred again (Government statements No 403 and 404/KPTS/PK.300/M/05/2022) [5].

FMD is characterized by several clinical signs, including fever, hypersalivation, and blisters on the oral mucosa, nose, and feet. These blisters can rupture, leading to swollen coronary bands [6,7]. The disease is highly infectious, with a morbidity rate of 100%, but it generally has a low mortality rate of 2%. However, the mortality rate can rise to 20% in young calves due to myocarditis [8]. In addition, FMD has a significant impact on the rural economy [9,10]. The cattle affected by FMD showed several symptoms that differ from one case to another depending on breed, the strain of the virus, and host immunity, with the main clinical symptoms blistering in the mouth and on the foot, hypersalivation, vesicle on the nose and tongue and decrease appetite [11,12].

Scientific evidence has long established that adequate nutrition is crucial for bolstering resistance against infectious diseases, thereby facilitating an efficient immune response in livestock [13,14], production, and reproduction [15]. However, in actual practice, livestock ruminant raising by smallholder farmers typically provides limited quality forage, especially during the dry season [16]. As a result, it is necessary to improve the ration of the cattle with feed which is rich in protein and energy and has a high digestion value [17,18]. Bali cattle are vital to many Indonesians, but data on their response to FMD and feed availability during the dry season is scarce. Previous studies have shown that feed supplementation improves the production and reproduction of Bali cattle [19].

In recent years, in addition to substantial vaccination initiatives, numerous researchers have explored various nutritional approaches to combat FMD infection. These approaches include the utilization of antioxidant vitamins and microminerals [20–23], a natural antioxidant and phytochemical from the plant [24], enrichment of complete diets with Protelis^®^ concentrate [4,25], and the most recent one uses therapeutic diets [26]. However, many of these studies differ because the materials used are often expensive and difficult to afford at the farm level. In addition, most studies did not objectively evaluate the effects of their intervention on post-partum body condition score (BCS) recovery of the cow and calf weight gain response of treated dams after FMD infection.

The research aimed to evaluate the effect of nutrition-rich feed supplementation on the recovery enhancement of cows suffering from FMD, improved body condition, and effect on calf birth weight after recovery. Furthermore, the research result will be useful in providing a scientific-based solution to enhance the recovery of cattle affected by FMD. The quick recovery of cattle from the FMD effect could reduce the huge economic loss caused by FMD, and support the animal welfare and the sustainability of local farming.

## Materials and Methods

### Ethical approval

This research has been approved by the Research Ethics Committee of the Faculty of Animal Science, University of Mataram, and is thus following the ethical standards set out in the committee’s ethical approval number: 1903/FapetUN/ETIK/2023.

### Cattle and research management

The study was conducted during the dry season (late May to August). Thirty pregnant cows (approximately 7 months gestation, aged 4–5 years) were used. The cows belonged to a smallholder farmer and were kept in group housing, each tied to a post with a feeder and drinking bucket.

### Experimental design

The study employed a randomized complete block design with replicates as blocks. Treatments included: A) basal feed only (control), B) basal feed + concentrate 0.2% BW, C) basal feed + concentrate 0.4% BW, D) basal feed + concentrate 0.6% BW, and E) basal feed + concentrate 0.8% BW. Each treatment had six replicates, totaling 30 experimental units.

### Group distribution, treatment, and adaptation period

Cows were randomly assigned to five groups of six animals each. Group A served as the control (unsupplemented), while groups B, C, D, and E received concentrates at 0.2%, 0.4%, 0.6%, and 0.8% of body weight, respectively. The concentrate was a mix of cassava peels (60%), ground corn (40%), urea (3% of total feed), and minerals (2.5% of total feed). The concentrate used was a mixture of cassava peels (60%), ground corn (40%), urea (3% of total feed), and minerals (2.5% of total feed). The minerals used are SP Minerals produced by PT. Sumber Multi Vita, Jakarta, containing 500 IU of Vitamin A, 75,000 IU of Vitamin D3, 200 mg of Vitamin E, 222,000 mg of Calcium, 160,000 mg of Phosphorus, 12,000 mg of Magnesium, 24,000 mg of Sodium, 15,000 Ferrous mg, Manganese 13,750 mg, Zinc 12,500 mg, Copper 1,800 mg, Iodine 87.5 mg, Selenium 25 mg, and Cobalt 25 mg.

The adaptation period to concentrate feed lasted 14 days starting mid-May 2022. An FMD outbreak occurred in early June, and all infected cattle were treated with B-complex vitamins and Oxytetracycline LA injections every 5 days.

### Nutrient composition of feedstuff

Feedstuff nutrient composition was analyzed per AOAC (2012) procedures. Samples were dried, milled, and analyzed for dry matter (DM), organic matter (OM), crude protein (CP), crude fiber (CF), and ether extract (EE). The nutrient content of forage and concentrates is shown in Table 1, and the nutrient content of each feed treatment is displayed in Table 2.

### Forage and concentrate intake calculation

The concentrate was administered before forage. Refusal concentrate was weighed to determine consumption. Forage was weighed in the morning after the cows finished the concentrate, and the next morning refusal forage was also weighed. Consumed amounts were determined by subtracting the residue from the provided feed [18].

### Measurement of body condition scores

BCS were determined following [27,28] procedures, with scores ranging from 1 to 5. Evaluations included the backbone, hips, shoulders, ribs, tail-head, and general body frame. BCS examinations were conducted 1 week before treatment, one week after FMD exposure, 1 week after calving, and 1 week after lactation.

**Table 1.** The nutrient content of forage and concentrates are given to the cattle in the present study.

Feedstuff	Nutrient composition (%)					
	DM	Ash	OM	EE	CF	CP
Forages	89.72	10.93	78.75	0.79	27.79	9.02
Concentrate	87.56	3.07	85.57	0.73	4.52	10.68

DM: dry matter; OM: organic matter; EE: ether extract; CF: crude fiber; CP: crude protein.

**Table 2.** Nutrient content of forage and concentrate each treatment.

Treatment groups	Nutrient composition (%)			
	DM	OM	CF	CP
A (control)	89.65	78.99	26.64	6.87
Treatment B	86.94	85.56	5.44	7.88
Treatment C	89.02	86.90	3.51	10.22
Treatment D	89.45	81.19	4.19	12.25
Treatment E	86.69	84.25	4.59	14.06

DM: dry matter; OM: organic matter; EE: ether extract; CF: crude fiber; CP: crude protein.

### Clinical signs and fmd recovery examination

Clinical signs and recovery from FMD were assessed by daily examinations of hypersalivation, nasal discharge, snout and nose erosion, tongue and lip erosion, foot swelling, refusal to eat, and return to normal feed consumption.

### Calf birth weight measurement

Calves were weighed immediately after birth and before suckling using a Moritz spring-dial hoist scale with 0.1 kg sensitivity.

### Data analysis

All data were calculated and subjected to analysis using R software version 4.4.0 with the “agricolae” library in the CRAN package [29]. Significance was set at *p <* 0.05. The statistical model used was:

Yij = µ + αi + βj + εij,

where yij represents the observed value of each individual, µ denotes the overall mean, αi represents the treatment effect, βj are the block effects (replicates), and εij denotes the residual error (variation among replicates for each treatment). Moreover, data regarding BCS, clinical signs and FMD recovery, and birth weight of calves were presented in graphs and evaluated differences between supplementation treatments.

## Results

### Dry matter intake and concentrate feed intake

Our current results show that feed consumption did not differ among the treatment groups. However, concentrate intakes showed significant differences (*p* < 0.05). Numerically, feed consumption shows a slight increase in groups B and C, with optimal effect at 0.4% BW (group C). This indicates a slight increase in dry matter consumption from the administration of concentrates in groups A, B, and C, as much as 0%, 2%, and 0.4% of body weight.

Among the concentrate treatment groups, group E produced the highest concentrate intake compared to the other groups (2.13 kg/day) (*p <* 0.05). Group C for a concentrate intake of 1.08 kg/day and Group D for a concentrate intake of 1.39 kg/day did not show different responses on concentrate intake, but both were higher than group B (0.60 kg/day) (*p* *<* 0.05).

### FMD clinical responses

The results of clinical signs of FMD were observed 2 days after infection. These signs are the duration of hypersalivation, skin, tongue, and lips erosion, sores in the interdigital gaps, and lazy eating. The following observation was an increase in BCS and calf birth weight.

**Figure 1.**
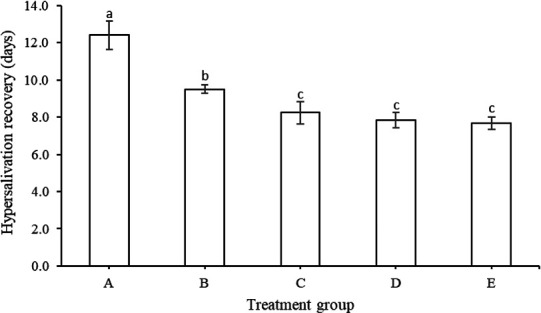
Duration of hypersalivation and nasal discharge (mean ± SE) in cows suffering from FMD with different feed supplementation.

Clinical signs of hypersalivation and nasal discharge in cattle suffering from FMD in the control (group A) were 12.4 days and shorter in group B (9.50 days) and group C (8.25 days) (*p *< 0.05). Furthermore, groups D and E were 7.83 days and 7.67 days, respectively, having the shortest day duration similar to group C (Fig. 1).

**Figure 2.**
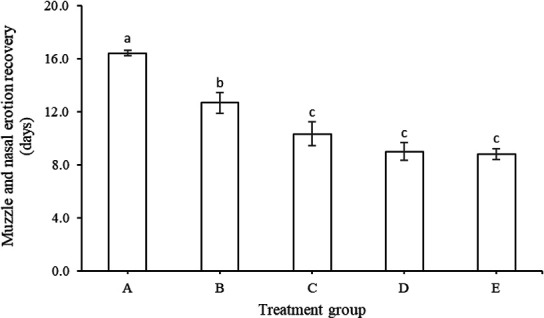
Duration of muzzle and nose erosion (mean ± SE) of cows suffering from FMD with different feed supplementation.

As for the blisters and erosion of the muzzle and nose of the cows suffering from FMD (Fig. 2) showed that the duration of duration of the exfoliation of the skin on the muzzle and nose, the blisters and erosions in all treatment supplementation groups healed faster than the control (*p <* 0.05). The duration of illness in group A was 16 days, while in treatment groups B, C, D, and group E returned to normal more quickly (12.6 days, 10.3, 9.00, and 8.83 days, respectively, *p <* 0.05).

**Figure 3.**
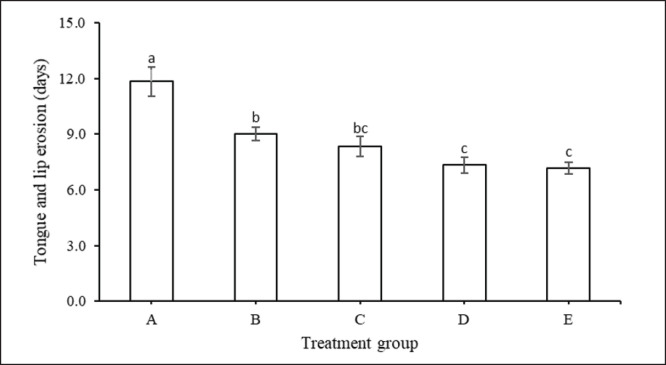
Duration of tongue and lip erosion (mean ± SE) of cows suffering from FMD with feed supplementation.

The study showed that the duration of the tongue and lips, abrasion, and erosion were significantly affected by the rich-feed supplementation treatment (*p <* 0.05). The examination of the tongue and lips, abrasion, and erosion in groups A, B, C, D, and E were 11.83, 9.83, 8.33, 7.33, and 7.17 days, respectively. Cows treated with the highest feed supplement (treatment E) produced a shorter duration of clinical symptoms of tongue and lip erosion compared to treatment group B and the control treatment group (A; with no supplementation) (*p <* 0.05). However, this group (E) did not show any difference in response with treatment groups C and D (Fig. 3).

**Figure 4.**
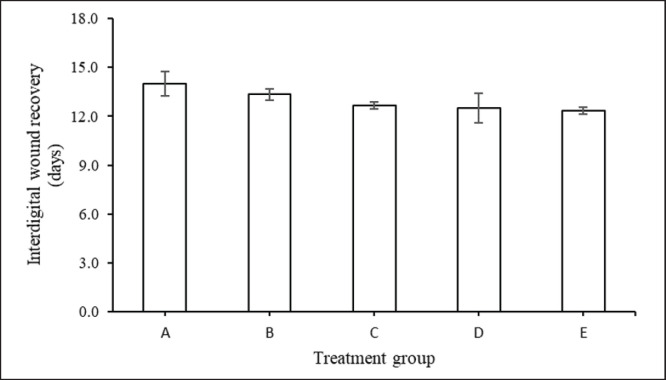
Duration of time (days) (mean ± SE) for cows suffering from FMD interdigital injuries with feed supplementation.

**Figure 5.**
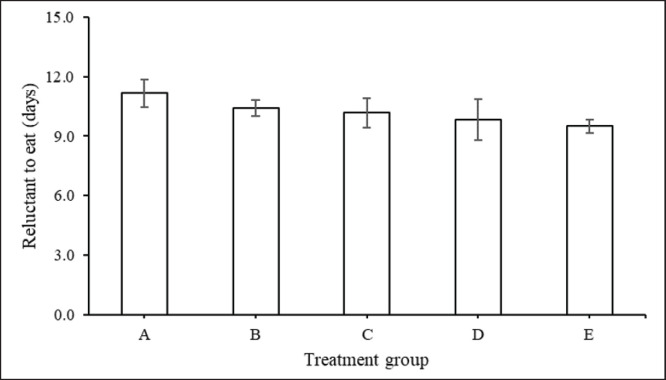
Duration of reluctant to eat returns to normal (mean ± SE) in cows suffering from FMD with feed supplementation.

Meanwhile, the addition of feed supplement does not affect the duration of interdigital wounds in cattle suffering from FMD (Fig. 4). The length of interdigital wounds in groups A, B, C, D, and E were 14.0, 13.3, 12.7, 12.5, and 12.3 days, respectively. Likewise, the case with reluctance to eat shows the same thing, and there is no difference in response among all treatment groups (Fig. 5). Although numerically, the duration of reluctant to eat in group A was 11.2 days, longer than groups with nutrient-rich supplementation, namely 10.4 days (group C), 10.2 days (group D), 9.83 (group E), and 9.50 days (group B).

### Body condition scores and calf birth weight

**Figure 6.**
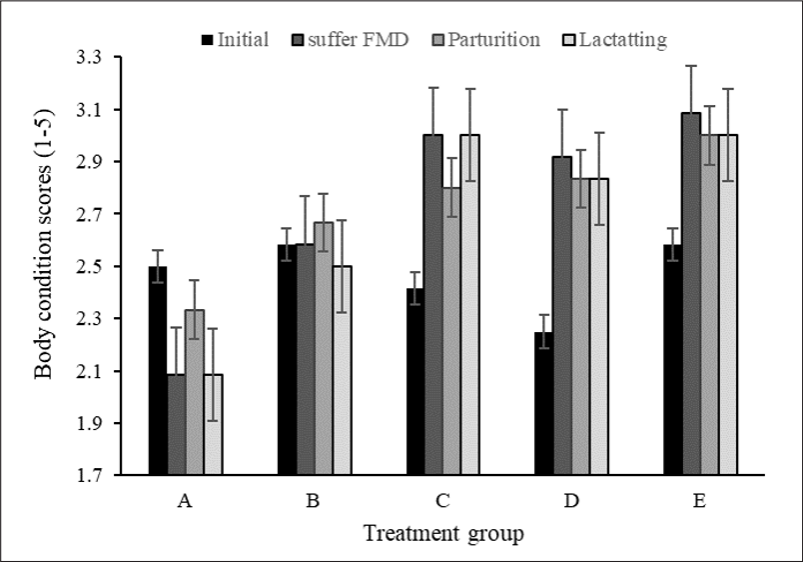
Body condition score (BCS) (mean ± SE) of cow initials, after suffering FMD, post-partum and during lactation, with concentrate supplementation, in the control (A) and treatment (B, C, D, and E) groups.

Feed supplementation at least maintained and even improved BCS post-FMD, post-calving, and during lactation. Calf birth weights were higher in supplemented groups, indicating that adequate protein intake mitigated the impact of FMD on calf birth weight. Figure 6 shows that feeding nutrient-rich feeds can shorten the time, allowing cows that refuse to eat to consume feed again. Feed consumption in the control group (A) returned to normal after 11.2 days, while in groups B, C, D, and E, it was 8.50, 9.50, 9.50, and 8.67 consecutive days. After the cow has an appetite again, the cow will be healthy and normal again, after suffering from FMD. Body condition after suffering FMD increased significantly, after birth decreased slightly, and after lactation remained (Fig. 6).

**Figure 7.**
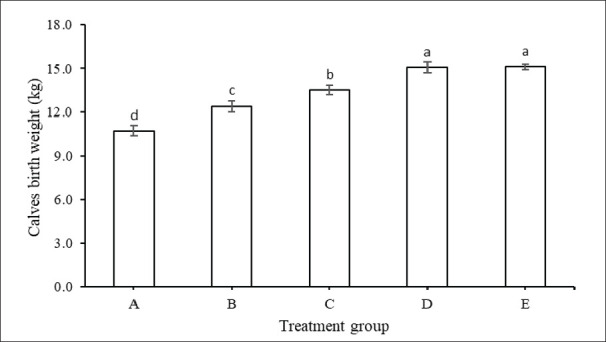
Calf birth weight (mean ± SE) of cows with feed supplementation from 7 months pregnancy in treatment groups A, B, C, D, and group E.

The calf birth weight in groups A, B, C, D, and E after the FMD outbreak are shown in Figure 7. Calf birth weight (kg) in groups A (control), B, C, D, and group E was increased concomitantly with the increase in the level of nutrient-rich feed supplementation (*p <* 0.05). The highest birth weight was obtained in group E, which yielded a birth weight of 15.1 kg and was successively followed by group D (15.1 kg), group C (13.5 kg), group B (12.4 kg), and group control (A) only produced a birth weight of 10.7 kg. Calf birth weight between groups E and D was not significantly different, but both showed significant differences compared to the remaining treatment groups (*p <* 0.05).

## Discussion

The precise timing of exposure to the FMD virus within this study remains undetermined. It has been documented that FMD transmission is highly rapid, with clinical symptoms manifesting within 3–5 days, though this period can vary between 2 and 14 days [8]. This study was conducted during the dry season, with limited feed quality and quantity [16,30]. Under these conditions, prolonged recovery was anticipated, prompting the improvement of feed quality through enriched protein and energy sources, as has been done by Kariyani et al. and Dahlanuddin et al. [18,31]. On the other hand, quality feed has been reported to accelerate the recovery of FMD by boosting immunity [32,33]. In another report, Saptahidhayat et al. [25] showed that a combination of king grass and Proteolis^®^ concentrate positively responded to clinical symptoms, antibodies, and milk production of dairy cows after FMD infection.

In our study, quality control of the supplemented with nutrient-rich feed was validated through proximate analysis, which revealed a consistent quality of forage and concentrate with minimal standard deviation throughout the trials (Table 1). Daily evaluation of concentrate intake for the treatment groups conformed precisely to the research plan, this underscores the effectiveness of the study approach in maintaining consistent concentrate quality. The concentrate materials were selected for their local availability, ease of procurement, and cost-effectiveness, aligning with recommendations for enhancing cattle productivity sustainably and profitably [34].

Dry matter intake (DMI) increased in groups A, B, and C, corresponding to 0%, 2%, and 0.4% body weight, respectively, indicating that concentrate supplementation effectively boosted dry matter consumption, reaching optimal levels in group C. The provision of concentrate provided better protein and energy available for digestion and also likely enhanced microbial activity in the rumen, thereby stimulating higher forage consumption and overall dry matter intake [35–37]. This aligns with previous findings that concentrate supplementation facilitates metabolic adaptation to sustain pregnancy [38].

Cattle with FMD infection experience mouth ulcers and anorexia, leading to reduced feed intake. As far as we understand, the provision of palatable concentrate feed in a finer and softer form is believed to increase intake. This corresponded with concentrate intake, which increased linearly in our study (Table 3). Although not directly related, similar results have also been shown by Somagond et al. [39], whose provision of a feeding mash total mixed ration high in energy and protein as a therapeutic diet can maintain feed intake and help to meet the dry matter requirements of male Friesian Holstein crossbreds. In addition, the increase in DMI in cattle-fed nutrient-rich supplementation was also due to the faster healing rate of tongue and lip erosion compared to the control group (Fig. 3).

Two weeks post-feed introduction, an FMD outbreak occurred, aligning with reports that FMD symptoms appear 1–3 days post-infection, with infection routes primarily through inhalation leading to viral replication in the mouth, pharynx, foot, and heart [8]. During the first period of the FMD outbreak, no commercial FMD vaccine was available in the country, making it impossible to control the disease through vaccination. Thus, the focus shifted to enhancing the natural immune response of livestock through nutritional support. Enhancing feed with better nutrition has been shown to improve immunity and overall health in livestock [32,40]. The immune system is known to naturally develop through adequate nutrition, which involves alterations in metabolism and increased glucose and fatty acid production. These nutrients are essential for the high energy requirements of the activated immune system during periods of infection. Immune cells adapt their energy sources by utilizing lipids, amino acids, and glucose. Activated lymphocytes primarily rely on aerobic glycolysis for ATP production when oxygen is available [41]. In our research, the feed is a formulated feed rich in other macro and micronutrients. Feeding a diet rich in energy, protein vitamins, and minerals such as zinc and selenium is essential for optimal immune function [42], as well as optimizing the recovery and healing process through modulating inflammatory and immune responses [43].

**Table 3.** Feed consumption and concentrate intake in the control (A) and treatment (B, C, D, and E) groups.

Variables	Treatment groups					sem	*p-*value
	A	B	C	D	E		
Feed consumtion (DMI, kg/day)	1.81 ± 0.11	1.87 ± 0.10	1.91 ± 0.16	1.85 ± 0.20	1.88 ± 0.12	0.062	0.864
Concentrate intake (kg/day)	0.00 ± 0.00^a^	0.60 ± 0.09^b^	1.08 ± 0.16^c^	1.39 ± 0.14^c^	2.13 ± 0.52^d^	0.112	<0.001

^a,b,c,d^ means in the same row with different superscripts differ significantly (*p <* 0.05).

A: without supplementation (control); B: 0.2% concentrate supplemented; C: 0.4% concentrate supplemented; D: 0.6% concentrate supplemented; E: 0.8% concentrate supplemented; sem: standard error of the means.

Remarkably, all 30 cows in this study recovered despite a 100% morbidity rate, with no fatalities. This contrasts with reported FMD mortality rates of up to 2% [8]. The rapid recovery observed may be attributed to the natural immunity and resilience of Bali cattle, alongside the benefits of concentrate supplementation, which enhances rumen microbial efficiency and digestion [35]. This highlights that proper nutrition significantly contributes to improved immunity and general health of cattle. The immune system consists of various components crucial for protecting animals from diseases, with the adaptive immune system focusing on preventing infections, eliminating existing ones, and restoring tissue health [44].

Early symptoms of FMD, such as hypersalivation and nasal discharge, are seen after the onset of fever. Research findings show that concentrate supplementation shortens the duration of all FMD lesions (Figs. 1–5) such as discharge from the mouth and nose, erosion of the snout and nose, erosion of the tongue and lips, sores in the digital space, and increased appetite. Healing and recovery can be attributed to a rich feed supply of nutrients including protein, through the blood. Hence, increases immunity and defense against FMD, as reported by Grubman and Baxt [45].

Concentrate supplements also speed up the return of appetite to normal and can speed up the return from 11.2 to 9.5 days (Fig. 5). Improving concentrate quality (Table 2) contributes to faster appetite recovery and overall health recovery [38]. In this particular instance, the hypothesis can be advanced that the concentrate contains amounts of rapidly fermentable non-structural carbohydrates and other micronutrients. These elements, it is suggested, may have engendered optimal conditions conducive to rumen fermentation. The fermentation of concentrate in the rumen results in the production of propionic acid, which serves as a precursor for glucose in ruminants [36,46]. The glucose produced will then fulfill the need for energy, which in turn triggers a hormonal response that leads to a naturally formed immune system. Thus, the sufficiency of these nutrients may have facilitated the accelerated recovery process, a phenomenon previously discussed in other sections of the present study.

The condition of the cow, as assessed by the BCS, reflects the adequacy of the better feed to meet the cow’s physiological needs during the recovery period from illness, the post-partum period, and the lactation period [27,28]. In this study, in the control group (A), BCS decreased after FMD. In groups B, C, D, and E, supplementary feed with increased protein serially caused BCS to stabilize or improve, even after FMD infection (Fig. 6). Improving the quality of feed intake and digestibility from concentrate supplementation is essential to maintain BCS, especially during pregnancy and lactation [47]. The apparent increase in BCS was shown in A, B, and C groups. The highest increase in BCS was found in group C, with a CP of 10% (Table 2), while in groups D and E even though achieved the highest BCS the increase was lower compared to group C did not experience an increase (Fig. 6).

Birth weight was higher in the supplemented group than in controls, with a significant increase in groups C and D, indicating a positive impact of a protein-rich diet on calf birth weight despite exposure to FMD [48]. High-quality feed appears to reduce the adverse effects of FMD on pregnant cows and their calves. Calf birth weight is related to BCS and general cow health, especially during pregnancy. A higher cow’s BCS is correlated with higher calf growth in utero [49]. This research shows that increasing feed quality aligns with increasing calf birth weight. These results align with previous results in Bali cattle, which show that feed quality increases BCS [48].

This study confirms that concentrate supplementation can significantly expedite recovery from FMD, enhance BCS post-illness, post-partum, and during lactation, and improve calf birth weight. These findings consistently show that concentrate supplementation boosts cattle productivity and reproductive performance [50]. Additionally, concentrate feed supplements during the dry season increase oestrus rates and pregnancy success [51], supporting overall cattle health and productivity [17].

It is acknowledged that further research is necessary to substantiate the efficacy of nutrient-rich supplementation in Bali cattle. Such research should encompass an evaluation of the immune system and its physiological status and assessing the reduction in FMD virus incidence. Regrettably, we cannot test these variables now due to resource constraints. Further research is necessary to substantiate the positive response of nutrient-rich feed supplementation in combating FMD diseases, with the overarching objective being to gain profound insight into the physiological mechanisms involved.

## Conclusion

This study demonstrates that nutrient-rich feed supplementation significantly enhances the recovery of Bali cattle affected by FMD. Supplementation with concentrates effectively increased dry matter intake and accelerated the cessation of clinical signs such as hypersalivation, nasal discharge, muzzle and nose erosion, and inter-digital wounds. Additionally, supplementation improved the BCS of the cows post-FMD, post-partum, and during lactation. Calf birth weights also increased with higher protein levels in the diet, indicating that adequate nutrition can mitigate the adverse effects of FMD on pregnant cows and their offspring. Among the different levels of supplementation, a concentrated level of 0.6% of body weight daily was found to be the most effective in promoting recovery and maintaining optimal body condition and birth weight.

## List of abbreviations

BCS, Body condition scores; CF, Crude fiber; CP, Crude protein; DM, Dry matter; FMD, Foot and mouth diseases; OM, Organic matter.

## References

[1] Khan D, Sheikh IS, Ullah A, Kasi KK, Mustafa MZ, Din ZU (2024). Circulation of foot-and-mouth disease serotypes, risk factors, and their effect on hematological and biochemical profiles among cattle and buffalo in Quetta, Balochistan, Pakistan. Vet World.

[2] Bradhurst RA, Roche SE, East IJ, Kwan P, Garner MG (2015). A hybrid modeling approach to simulating foot-and-mouth disease outbreaks in Australian livestock. Front Environ Sci.

[3] Mashinagu MM, Wambura PN, King DP, Paton DJ, Maree F, Kimera SI (2024). Challenges of controlling foot-and-mouth disease in pastoral settings in Africa. Transbound Emerg Dis.

[4] Ranjan R, Biswal JK, Sharma GK, Pattnaik B (2016). A review on foot-and-mouth disease: pathology, diagnosis and its management. Indian J Vet Pathol.

[5] Ditjen PKH (2023). Informasi penanggulangan dan tindakan pencegahan wabah PMK. https://SiagapmkCrisis-CenterId/IndexPhp.

[6] Lyons NA, Alexander N, Stärk KDC, Dulu TD, Sumption KJ, James AD (2015). Impact of foot-and-mouth disease on milk production on a large-scale dairy farm in Kenya. Prev Vet Med.

[7] Aslam M, Alkheraije KA (2023). The prevalence of foot-and-mouth disease in Asia. Front Vet Sci.

[8] Azeem A (2020). A review on foot and mouth disease in dairy animals, etiology, pathogenesis and clinical findings. Pure Appl Biol.

[9] Knight-Jones TJD, Rushton J (2013). The economic impacts of foot and mouth disease - What are they, how big are they and where do they occur?. Prev Vet Med.

[10] Sahara, Sugema I, Amaliah S, Probokawuryan M, Ahmad FS (2023). Assessing the impacts of food and mouth disease outbreak on the Indonesian economy and its regional growth. J Manag Agribus.

[11] Nampanya S, Khounsy S, Phonvisay A, Young JR, Bush RD, Windsor PA (2015). Financial impact of foot and mouth disease on large ruminant smallholder farmers in the Greater Mekong Subregion. Transbound Emerg Dis.

[12] Jumaa RS, Mohsin SI, Abdulmjeed DI, Atshan OF (2021). Foot and mouth disease virus: a review. Magna Sci Adv Biol Pharm.

[13] Hamidianshirazi M, Ekramzadeh M, Hamidianshirazi AR, Zangene A (2022). Association between nutrition and immune system: a review. Int J Nutr Sci.

[14] Williams AR, Andersen-Civil AIS, Zhu L, Blanchard A (2020). Dietary phytonutrients and animal health: regulation of immune function during gastrointestinal infections. J Anim Sci.

[15] Izquierdo AC, Reyes AEI, Lang GR, Oaxaca JS, Liera JEG, Mancera EA V (2021). Nutrition and food in the reproduction of cattle. Eur J Agric Food Sci.

[16] Sutaryono YA, Putra RA, Mardiansyah, Yuliani E, Harjono, Mastur (2023). Mixed *Leucaena *and molasses can increase the nutritional quality and rumen degradation of corn stover silage. J Adv Vet Anim Res.

[17] De Faria AC, Bolson DC, Dos S Pina D, Prado TA, Roecker AN, Chaves CS (2024). Intensively reared nelore steers with levels of concentrate and protein sources during the dry season. Animals.

[18] Kariyani LA, Dahlanuddin, Panjaitan T, Putra RA, Harper K, Poppi D (2021). Increasing the level of cassava chips or cassava pilp in leucaena based diets increases feed intake and live weight gain of Bali bulls. LRRD.

[19] Dahlanuddin, Panjaitan T, Waldron S, Halliday MJ, Ash A, Morris ST (2019). Adoption of *leucaena*-based feeding systems in Sumbawa, eastern Indonesia and its impact on cattle productivity and farm profitability. Trop Grassl-Forrajes Trop.

[20] Yörük IH, Tanritanir P, Dede S, Ceylan E, Ragbetli C (2014). Antioxidant vitamins and microminerals in cows with foot-and-mouth diseas. Indian J Anim Res.

[21] EL-Bayoumi MK, Abdelrahman KA, Farag TK, Allam AM, Abou-zeina HAA (2014). Zinc , vitamin E and selenium oral supplementation reduces the severity of foot-and-mouth disease clinical signs in sheep. Glob Vet.

[22] Zhao XJ, Li ZP, Wang JH, Xing XM, Wang ZY, Wang L (2015). Effects of chelated Zn/Cu/Mn on redox status, immune responses and hoof health in lactating Holstein cows. J Vet Sci.

[23] Abou-Zeina HAA, Nasr SM, Nassar SA, Farag TK, El-Bayoumy MK, Ata EB (2019). Beneficial effects of antioxidants in improving health conditions of sheep infected with foot-and-mouth disease. Trop Anim Health Prod.

[24] Vougat R, Foyet H, Garabed R, Ziebe R (2015). Antioxidant activity and phytochemical constituent of two plants used to manage foot and mouth disease in the Far North Region of Cameroon. J Intercult Ethnopharmacol.

[25] Saptahidhayat N, Airin CM, Yanuartono, Widiasih DA, Indarjulianto S, Irianingsih SH (2023). Rejuvenated of dairy cows after foot and mouth disease infection using combination of complete feed to increase milk production. Adv Anim Vet Sci.

[26] Somagond A, Patel BHM, Pattanaik AK, Krishnaswamy N, Mahadappa P, Singh M (2024). Evaluation of feeding different forms of therapeutic diet on the feed intake, digestibility, feed efficiency, and growth of calves experimentally infected with foot-mouth disease virus. Vet Res Commun.

[27] Soares FS, Dryden GM (2011). A body condition scoring system for Bali cattle. Asian-Australas J Anim Sci.

[28] Burrow H (2019). Strategies for increasing beef cattle production under dryland farming systems. Indon Bull Anim Vet Sci.

[29] R Core Team (2022). R: A language and environment for statistical computing.

[30] Sutaryono YA, Supriadi D, Imran, Putra RA (2019). Seasonal growth of *Leucaena leucocephala* cv. Tarramba in dry land of west Sumbawa, Indonesia. Trop Grassl-Forrajes Tropicales.

[31] Dahlanuddin, Kariyani LA, Panjaitan TS, Putra RA, Harper KJ, Poppi DP (2024). Growth rate of male Bali cattle (*Bos javanicus*) fed leucaena and rice straw diets with increasing levels of cassava. Anim Prod Sci.

[32] Ingvartsen KL, Moyes K (2013). Nutrition, immune function and health of dairy cattle. Animal.

[33] Edirisinghe N, Flavel M, Pouniotis D, Zakaria R, Lim KF, Dias DA (2024). From feed to fork: immunity, performance and quality of products from farm animals fed sugarcane products. Front Anim Sci.

[34] Selemani IS, Eik LO (2016). The effects of concentrate supplementation on growth performance and behavioral activities of cattle grazed on natural pasture. Trop Anim Health Prod.

[35] Rocha TC, De Alencar Fontes CA, Da Silva RTS, Processi EF, do Valle FRAF, Lombardi CT (2016). Performance, nitrogen balance and microbial efficiency of beef cattle under concentrate supplementation strategies in intensive management of a tropical pasture. Trop Anim Health Prod.

[36] Dilaga SH, Putra RA, Pratama ANT, Yanuarianto O, Amin M, Suhubdy. (2022). Nutritional quality and *in vitro* digestibility of fermented rice bran based on different types and doses of inoculants. J Adv Vet Anim Res.

[37] Dilaga SH, Putra RA, Sofyan, Yanuarianto O, Amin M (2022). Pengaruh sumber energi yang berbeda dalam formulasi pakan terhadap pertumbuhan pedet jantan sapi bali lepas sapih. J Triton.

[38] Prezotto LD, Thorson JF (2023). Effect of dietary urea in gestating beef cows: circulating metabolites, morphometrics, and mammary secretions. Animals.

[39] Somagond A, Patel BHM, Pattanaik AK, Dutt T, Sanyal A, Sheshagiri G (2022). Economic evaluation of therapeutic diet formulated for foot and mouth disease (FMD) infected crossbred calves. Indian J Anim Sci.

[40] Montout L, Poullet N, Bambou JC (2021). Systematic review of the interaction between nutrition and immunity in livestock: Effect of dietary supplementation with synthetic amino acids. Animals.

[41] Bobeck EA (2020). Nutrition and health: companion animal applications: functional nutrition in livestock and companion animals to modulate the immune response. J Anim Sci.

[42] Kegley EB, Ball JJ, Beck PA, Bill E (2016). Kunkle interdisciplinary beef symposium: impact of mineral and vitamin status on beef cattle immune function and health. J Anim Sci.

[43] Tipton KD (2015). Nutritional support for exercise-induced injuries. Sports Med.

[44] Sordillo LM (2016). Nutritional strategies to optimize dairy cattle immunity. J Dairy Sci.

[45] Grubman MJ, Baxt B (2004). Foot-and-mouth disease. Clin Microbiol Rev.

[46] Mudhita IK, Putra RA, Rahman MM, Widyobroto BP, Agussalim, Umami N (2024). The silage quality of pennisetum purpureum cultivar gamma umami mixed with *Calliandra calothyrsus *and *Lactiplantibacillus plantarum*. Trop Anim Sci J.

[47] Jardstedt M, Hessle A, Nørgaard P, Frendberg L, Nadeau E (2018). Intake and feed utilization in two breeds of pregnant beef cows fed forages with high-fiber concentrations. J Anim Sci.

[48] Dahlanuddin, Yulianto TB, Priyanti A, Poppi DP, Quigley SP (2012). Weaning and supplementation increase liveweight Gain of Bali (*Bos javanicus*) cattle of small-holder farmers in Central Lombok, Indonesia. Anim Prod.

[49] Linden TC, Bicalho RC, Nydam DV (2009). Calf birth weight and its association with calf and cow survivability, disease incidence, reproductive performance, and milk production. J Dairy Sci.

[50] Santos MER, Santos A de D, da Fonseca DM, Sousa BM de L, Gomes VM, De Sousa DOC (2016). Cattle production supplemented on signal grass pastures during the rainy season. Acta Sci Anim Sci.

[51] Khlil ZB, Khnissi S, Rekik M, Lassoued N (2017). Feed supplementation improves estrus response and increases fertility of sheep induced to breed out of season. Trop Anim Health Prod.

